# Changes of corneal high-order aberrations after femtosecond laser-assisted in situ keratomileusis

**DOI:** 10.1097/MD.0000000000010618

**Published:** 2018-05-04

**Authors:** Jing Wang, Yanlin Ren, Kun Liang, Zhengxuan Jiang, Liming Tao

**Affiliations:** Department of Ophthalmology, the Second Affiliated Hospital of Anhui Medical University, Hefei, P.R. China.

**Keywords:** femto-lasik, high-order aberration cornea, myopia

## Abstract

**Background:**

Femtosecond laser-assisted in situ keratomileusis (FS-LASIK) has gained widespread popularity as a safe, effective and predictable treatment for correcting myopia and myopic astigmatism.However, complications such as biomechanical changes, structural weakness, dry eye and induction of high-order aberrations (HOAs) have been associated with FS laser excision. The induction of HOAs has been reported to reduce quality of vision, leading to increased glare, halos, starburst and deterioration of contrast function corneal HOAs play a significant role in whole-eye aberration. Thus, it is necessary to investigate the changes of corneal high-order aberrations after FS-LASIK.

**Methods:**

One hundred thirty-four eyes from 68 consecutive patients with myopia or myopic astigmatism were enrolled in this study. Corneal topography and visual acuity were measured preoperatively and at 1, 3, 6, and 12 months after FS-LASIK. Wavefront errors from the whole cornea, anterior cornea, and posterior cornea were measured by Pentacam.

**Results:**

Corneal aberrations on the posterior surface were less affected by FS-LASIK compared with those on the anterior surface and the whole cornea. The high-order aberrations (HOAs) on the whole and anterior corneal surfaces increased significantly at 1 month after surgery (*P* = .000, *P* = .000), while HOAs on the posterior surface did not significantly change (*P* = 1.000). The spherical aberration on the whole corneal and anterior corneal surfaces were significantly increased at 1 and 3 months postoperatively (*P* = .000 and *P* = .000, respectively), along with the vertical coma on the whole and anterior corneal surfaces at 1 and 3 months (*P* = .000 and *P* = .000, respectively). There was no significant difference in horizontal coma or trefoil on the whole, anterior and posterior corneal surfaces after surgery compared with preoperatively (all *P* = 1.000).

**Conclusion:**

After FS-LASIK changes in corneal aberration occurred mainly on the anterior surface, which may have a significant effect on visual quality.

## 1 Introduction

Femtosecond laser-assisted in situ keratomileusis (FS-LASIK) has gained widespread popularity over the last several decades as^[1–3]^ a safe, effective, and predictable treatment for correcting myopia and myopic astigmatism. With the progress of refractive surgery, patients’ expectations for refractive surgery are limited to visual acuity but to visual quality as well.^[4,5]^

The femtosecond (FS) laser is a regulatory invention for flap creation designed to improve patients’ quality of vision.^[6]^ The FS laser allows the surgeon to more accurately cut tissue and create stromal flaps for laser-assisted in situ keratomileusis (LASIK). The accuracy of the FS laser also helps maintain the morphology of the cornea. However, complications such as biomechanical changes, structural weakness, dry eye, and induction of high-order aberrations (HOAs) have been associated with FS laser excision.^[7]^

The induction of HOAs has been reported to reduce quality of vision^[8]^, leading to increased glare, halos, starbursts,^[9,10]^ and deterioration of contrast function.^[11,12]^ It has been reported that wavefront corneal aberrations are less likely with the FS laser compared with the mechanical microkeratome,^[13]^ which has led to increased acceptance of FS-LASIK over recent years.

Gobbe et al^[14]^ have reported that corneal HOAs play a significant role in whole-eye aberrations. Thus, it is necessary to investigate the changes of corneal high-order aberrations after FS-LASIK. In this retrospective study, we use the rotating Scheimpflug tomography system to measure HOAs on different corneal surfaces before and after FS-LASIK.

## 2 Materials and methods

### 2.1 Subjects

One hundred thirty-four eyes from 68 patients (31 men and 37 women) with myopia and myopic astigmatism were analyzed in this retrospective study. Patients attended the refractive surgery service at the Second affiliated Hospital of Anhui Medical University in China from June 2013 to February 2015. The study was approved by the investigational review board at the university. All procedures complied with the tenets of the Declaration of Helsinki, as revised in 2000. Informed consent was obtained from each patient. Inclusion criteria included the following: age 18 to 39 years, stable manifest refraction for the past 24 months, gas-permeable contact lens use discontinued for at least 4 weeks and soft contact lens use discontinued for at least 2 weeks. Exclusion criteria included the following: ophthalmic disease including prior corneal abnormalities and trauma, systemic disease including diabetes mellitus and connective tissue disease known to affect the eye, and a known history of ocular surgery.

Each patient had a rigorous screening examination that included uncorrected distance visual acuity (UDVA), corrected distance visual acuity (CDVA), slit-lamp examination, intraocular pressure (IOP), corneal topography, dynamic infrared pupillography, ocular wavefront analysis, and dilated fundus examination. The anterior, posterior, and total corneal aberrations were measured using the rotating Scheimpflug tomography system before surgery and at 1, 3, 6, and 12 months postoperatively.

Before the refractive surgery, informed consent was obtained for all patients after explaining the nature of the study and the benefits and risks of FS-LASIK. The study adhered to the Declaration of Helsinki and was approved by the Institutional Review Board of the Second affiliated Hospital of Anhui Medical University.

### 2.2 FS-LASIK procedure

All patients received FS-LASIK from an experienced surgeon. The cornea was pre-anesthetized with oxybuprocaine eye drops, and 2% sodium hyaluronate solution was placed on the cornea before applying the laser hand piece and suction ring. A 500 kHz FS laser system (Ziemer LDV, Switzerland) was used to create the flap. All flaps had a superior hinge, and the intended flap diameter and thickness were 8.5 mm and 100 μm, respectively. The patient was asked to stare at the internal fixation light, and the surgeon adjusted the position of the patient's eye in relation to the patient interface. Once the patient interface was focused on the pupil, the surgeon activated the suction mechanism incorporated in the patient interface. Ablation was then performed using the AMARIS 500 Hz excimer laser (SCHWIND AMARIS eye-tech-solutions, Germany). The stromal bed was flushed with a balanced salt solution, and the flap was then replaced.

### 2.3 Measurement of corneal wavefront aberrations

The aberrations of the whole, anterior and posterior cornea were measured using the rotating Scheimpflug tomography system (Pentacam, Oculus, Germany). This equipment includes a rotating camera that takes 50 images in 2 seconds, and each image contains 500 true elevation points. These elevation data can produce a three-dimensional reconstruction of the cornea, and the internal software can automatically transform the data points into corneal wavefront data using Zernike polynomials. All Zernike coefficients and root mean square values were calculated for a pupil diameter of 6.0 mm. Previous studies have shown that the Pentacam offers accuracy and precision in measuring corneal wavefront errors.^[15,16]^ Each patient was measured preoperatively and at 1, 3, 6, and 12 months postoperatively. The images measured with the Scheimpflug device were only used if the data quality statement was defined as OK.

### 2.4 Statistical analysis

Statistical analysis was performed using SPSS 20.0 software (IBM Singapore Pte Ltd, Singapore), and the Bonferroni test was used to obtain the time-development changes in the corneal wavefront aberrations preoperatively and at 1, 3, 6, and 12 months postoperatively. Data were recorded as mean ± standard deviation, and visual acuity results were converted to a logarithm of the minimum angle of resolution (logMAR). A *P*-value of <.05 was considered statistically significant.

## 3 Results

This retrospective study contains a total of 134 eyes from 68 patients (31 men and 37 women with mean age 24.99 ± 5.24 years) with myopia and/or myopic astigmatism.

### 3.1 Visual and refractive data

As demonstrated in Table 1, there was a statistically significant reduction of spherical equivalent (SE) at 1 month (*P* = .000), with no significant reduction after the 1-month follow-up. As demonstrated in Table 2, the logMAR UDVA improved significantly from 1.17 ± 0.35 preoperatively to –0.008 ± 0.37 at 12 months postoperatively (*P* = .000).

**Table 1:**
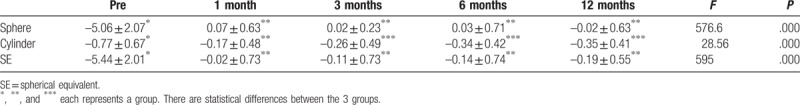
Mean sphere, cylinder, and SE data preoperatively and at 1, 3, 6, and 12 months postoperatively.

**Table 2:**
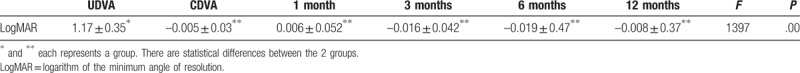
Uncorrected distance visual acuity (UDVA) and corrected distance visual acuity (CDVA) preoperatively and at 1, 3, 6, and 12 months postoperatively.

### 3.2 Wavefront aberrations after FS-LASIK

**Figure 1:**
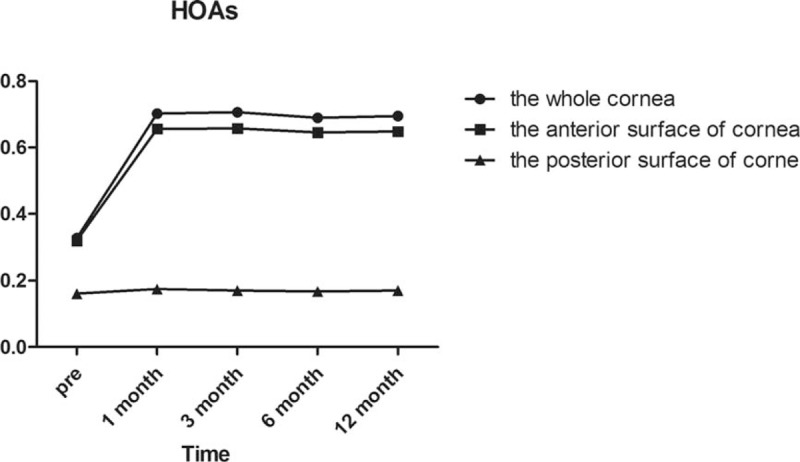
Wavefront aberrations of the whole, anterior, and posterior corneal surfaces at 6 mm in diameter preoperatively and at 1, 3, 6, and 12 months postoperatively.

As demonstrated in Fig. 1 and Tables 3–5, HOAs of the whole and anterior corneal surfaces were significantly increased at 1 month compared with preoperatively (*P* = .000 and *P* = .000, respectively). Meanwhile, there were no significant changes at 3, 6, and 12 months. The Pearson correlation coefficient of HOAs between anterior and whole corneal aberrations was 0.995 (*P* = .000), while the Pearson correlation coefficient of HOAs between posterior and whole corneal aberrations was 0.335 (*P* = .000). The posterior surface of the cornea did not significantly change after surgery throughout the entire 12-month follow-up (*P* = 1.000). In the whole corneal surface high-order aberrations, we found that the horizontal coma and trefoil o before surgery and 1/3/6/12 months postoperatively were not statistically different according to Post hoc test as demonstrated in Table 5.

**Table 3:**
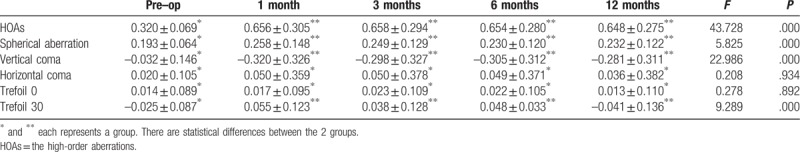
Anterior corneal surface.

**Table 4:**
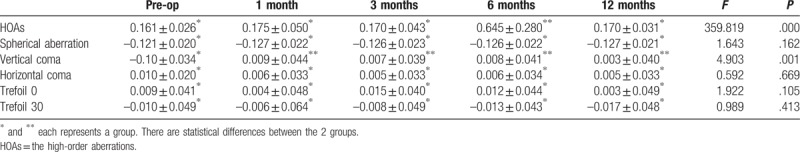
Posterior corneal surface.

**Table 5:**
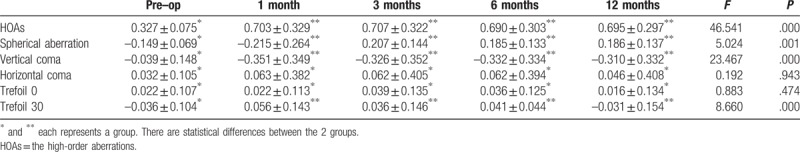
Whole corneal surface.

**Figure 2:**
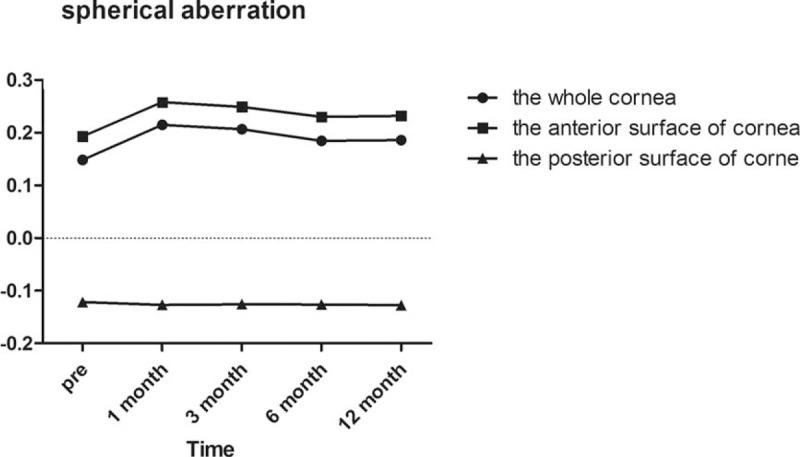
Spherical aberration of the whole, anterior, and posterior corneal surfaces at 6 mm in diameter preoperatively and at 1, 3, 6, and 12 months postoperatively.

Regarding spherical aberration as demonstrated in Fig. 2, there was a significant increase in the whole and anterior corneal surfaces at 1 and 3 months, postoperatively (all *P* = .000). However, there were no significant changes in the whole corneal surface at 6 and 12 months postoperatively (*P* = .263 and *P* = .119, respectively) or the anterior corneal surface at 6 and 12 months postoperatively (*P* = .219 and *P* = .077, respectively). Furthermore, the spherical aberration of the whole and anterior corneal surfaces began to gradually decline after 6 months, while the spherical aberration of the posterior corneal surface remained constant over time. Notably, the Pearson correlation coefficient between the anterior and whole corneal spherical aberrations was 0.990 (*P* = .000), and the Pearson correlation coefficient between the posterior and whole corneal spherical aberrations was 0.039 (*P* = .000).

**Figure 3:**
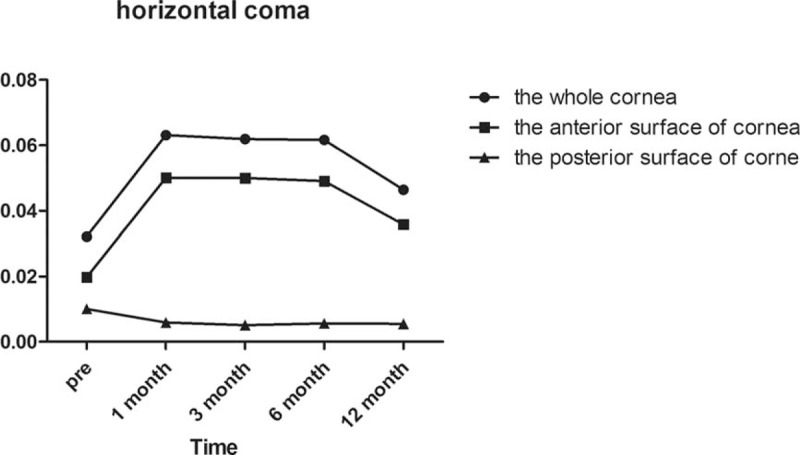
Horizontal coma of the whole, anterior, and posterior corneal surfaces at 6 mm in diameter preoperatively and at 1, 3, 6, and 12 months postoperatively.

**Figure 4:**
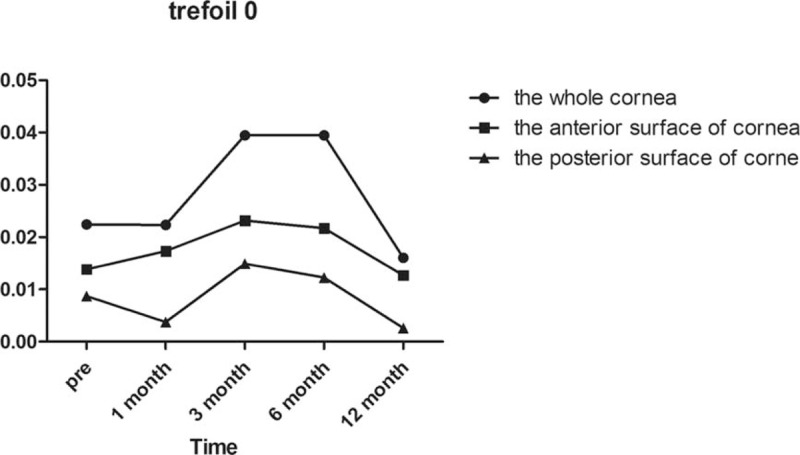
Trefoil 0 of the whole, anterior, and posterior corneal surfaces at 6 mm in diameter preoperatively and at 1, 3, 6, and 12 months postoperatively.

**Figure 5:**
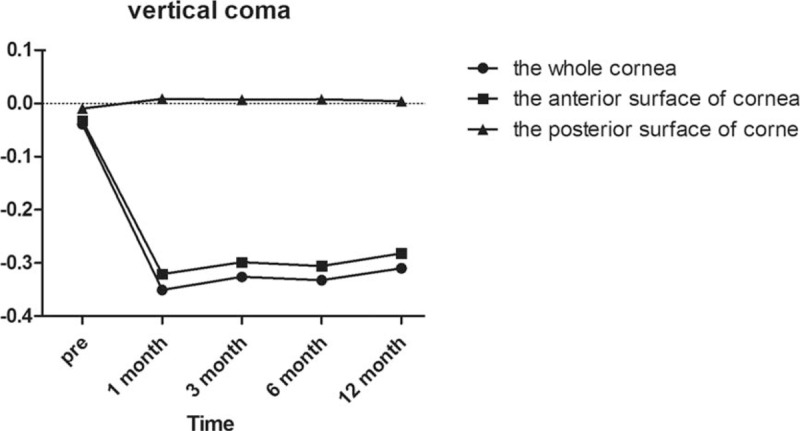
Vertical coma of the whole, anterior, and posterior corneal surfaces at 6 mm in diameter preoperatively and at 1, 3, 6, and 12 months postoperatively.

**Figure 6:**
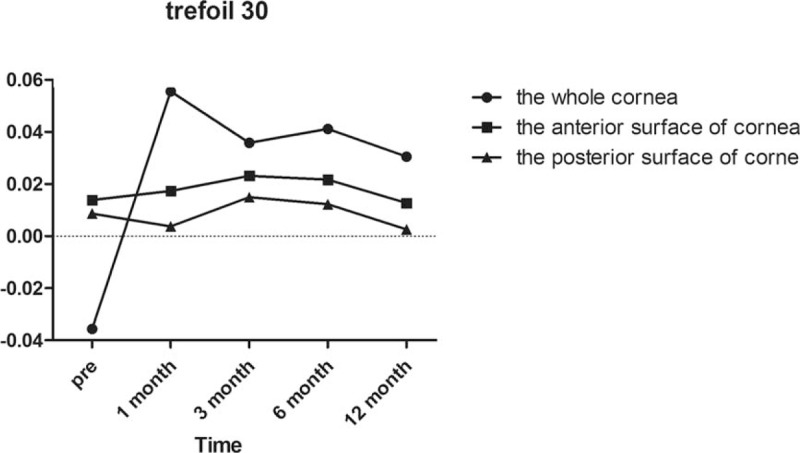
Trefoil 30 of the whole, anterior, and posterior corneal surfaces at 6 mm in diameter preoperatively and at 1, 3, 6, and 12 months postoperatively.

Regarding horizontal coma as demonstrated in Fig. 3, there was not a significant difference in the whole anterior or posterior corneal surfaces after surgery compared with preoperatively (all *P* = 1.000). In the anterior corneal surface high-order aberrations, we found that the horizontal coma and trefoil 0 before surgery and 1/3/6/12 months postoperatively were not statistically different according to Post hoc test as demonstrated in Table 3 and Fig. 4. However, there was a statistically significant increase in vertical coma on the whole and anterior corneal surfaces compared with preoperatively at 1 and 3 months (*P* = .000 and *P* = .000, respectively) as demonstrated in Table 3 and Fig. 5. Various high-order aberrations other than vertical coma before surgery and 1/3/6/12 months postoperatively on the posterior corneal surface were not statistically different according to Post hoc test as demonstrated in Table 4. And 6 months of higher-order aberrations were significantly different from the rest of the group. The Pearson correlation coefficient between anterior and whole corneal vertical coma was 0.996 (*P* = .000). The Pearson correlation coefficient between posterior and whole corneal vertical coma was –0.503 (*P* = .000). There was no significant difference in trefoil 0 on the whole, anterior and posterior corneal surfaces after surgery compared with preoperatively (all *P* = 1.000) as demonstrated in Fig. 4. Compared with before surgery, the trefoil 30 on the whole corneal surface at 1 month postoperatively was increased to 0.0556, although this was not statistically significant (*P* = 1.000) as demonstrated in Fig. 6.

## 4 Discussion

After FS-LASIK surgery, most patients gained satisfactory visual and refractive outcomes at 1 month postoperatively; 98% of eyes achieved ≥20/20 vision, and all patients achieved a refractive error within ±0.25 D of emmetropia, which remained constant over 12 months. However, laser refractive surgery can lead to significant visual disturbances, including double vision, glare, halos, starbursts, and a decrease in contrast sensitivity,^[17,18]^ which are induced by high-order aberrations generated by the cornea.^[19–21]^ In this retrospective study, we investigated time-dependent changes of high-order aberrations on the whole, anterior and posterior corneal surface before and after FS-LASIK surgery.

An aberration is the difference between an ideal image and a real image. An aberration causes the point of an image to appear as a fuzzy dispersion spot. The object of a flat image no longer appears to be in an object plane but rather appears as a curved surface with a loss of contrast sensitivity. A Zernike analysis is used to express ocular wavefront errors, and a root mean square is the criterion used to measure the magnitude of the wavefront aberration. Previous studies have reported a high correlation between an increase in corneal wavefront aberrations and the quality of vision after excimer laser surgery.^[22,23]^

For all patients in this study, we used an FS laser to create the corneal flap, cut the corneal collagen lamella, and then lifted and repositioned the corneal flap. This technique has been reported to induce high-order aberrations.^[24]^ Furthermore, the pupil offset from the visual axis can also induce high-order aberrations. Additional causes of high-order aberrations reported by other authors include wound response of the cornea after FS-LASIK, change in corneal shape after ablation, and individual differences in corneal biomechanical response.^[1,25]^

In this study, corneal high-order aberrations were increased, except in trefoil 0 and trefoil 30. According to our analysis, the increased rate of spherical aberration and coma after surgery were consistent with previous studies.^[26]^ Spherical aberrations and coma are the most important elements that affect vision quality. Former studies have shown a correlation between starburst and glare with spherical aberrations as well as a correlation between diplopia and horizontal coma.^[27]^ Thus, we focused on the changes in spherical aberrations and coma.

Our results demonstrate that the Pearson correlation coefficient of HOAs between the whole and posterior corneal aberrations was 0.335 (*P* = .000), while the Pearson correlation coefficient of spherical aberrations between the whole and posterior corneal surfaces was 0.039 (*P* = .000). However, there was not a significant correlation between HOAs on the whole and posterior corneal surfaces (*P* = 1.000). Previous studies^[28–31]^ reported that there were no significant changes in the posterior corneal elevation after FS-LASIK, which may correspond to our results demonstrating that there is no significant change in HOAs on the posterior corneal surface. However, the Pearson correlation coefficient between posterior and whole corneal vertical coma was –0.503 (*P* = .000), which may be explained by following patients longer than 12 months.

On the contrary, the Pearson correlation coefficient between anterior and whole corneal vertical coma was 0.996 (*P* = .000). The Pearson correlation coefficient of HOAs between anterior and whole corneal aberrations was 0.995 (*P* = .000). The Pearson correlation coefficient between anterior and whole corneal spherical aberrations was 0.990 (*P* = .000). The changes of HOAs, spherical aberration and vertical coma before and after surgery on the whole and anterior corneal surfaces increased at nearly the same rate. However, HOAs on the posterior corneal surface remained constant throughout the 12-month follow-up period. Furthermore, there were no significant changes in HOAs on the posterior corneal surface throughout the 12-month follow-up period. Thus, FS-LASIK primarily induces HOAs on the anterior corneal surface.

## 5 Conclusion

Our results extend our current understanding of the changes in corneal HOAs after FS-LASIK. We demonstrated that corneal HOAs mainly occurred on the anterior corneal surface. Since an increase in HOAs after FS-LASIK is associated with lower visual quality, it is important to investigate future solutions that will decrease the risk of HOAs after FS-LASIK and thus improve visual quality.

## Author contributions

**Conceptualization:** Liming Tao.

**Data curation:** Jing Wang, Yanlin Ren, Kun Liang, Zhengxuan Jiang.

**Formal analysis:** Jing Wang.

**Funding acquisition:** Liming Tao.

**Investigation:** Jing Wang, Yanlin Ren, Zhengxuan Jiang.

**Methodology:** Yanlin Ren.

**Project administration:** Jing Wang.

**Resources:** Kun Liang.

**Software:** Kun Liang.

**Validation:** Liming Tao.

**Writing – original draft:** Jing Wang.

**Writing – review and editing:** Liming Tao.
